# Clinical Group Students/Trainees Essay Contest Winner “Here Versus There: A Summer Vacation Story”

**DOI:** 10.4269/ajtmh.25-0533

**Published:** 2026-01-08

**Authors:** Ian Drobish, Jyotsna Nair, Hans Ackerman

**Affiliations:** ^1^ Physiology Unit, Laboratory of Malaria and Vector Research, National Institute of Allergy and Infectious Diseases, National Institutes of Health, Bethesda, Maryland;; ^2^ Critical Care Medicine Department, NIH Clinical Center, Bethesda, Maryland;; ^3^ Division of Infectious Diseases, Children’s National Hospital, Washington, DC

## Abstract

The American Committee on Clinical Tropical Medicine and Travelers’ Health’s Students/Trainees Leadership Group launched its essay contest in 2025 to encourage the involvement of emerging voices in tropical medicine in key issues shaping the field’s present and future.

The following authors won for their essays addressing the following prompt:

*“Tropical Medicine in the 21st Century”*

These essays were reviewed by a panel of clinician judges with experience in tropical medicine and writing, and the winning entries reflect both academic excellence and practical insight. Together, the two winning essays reveal the wide scope of tropical medicine, from policy and prevention to diagnosis and treatment, and the shared responsibility to confront these challenges wherever they arise.

## INTRODUCTION

“I don’t feel so well,” Monica said as she slumped back into her seat on the airplane. “You’ll feel better once those antibiotics have time to start working,” her mother replied. She sighed, and looked over at her younger brother, Michael, who was engrossed in an action movie, seemingly without a care in the world. Monica had just returned from the cramped airplane bathroom for what seemed like the tenth time. They were only a couple of hours into the flight, and she was counting down the minutes to landing, feeling particularly sorry for herself. Why did she have to be the only one in her family to get sick on the trip?

Just four weeks prior, at the beginning of August, Monica (age 13) and her brother, Michael (age 12), packed up with the rest of the family and got on a plane to visit their grandmother for the remainder of the summer break before returning to middle school in Montgomery County, MD, in early September. The trip had become a yearly tradition, and in addition to their immediate family, their extended family would typically join as well. For Michael and Monica, this meant countless hours hanging out with their many cousins, exploring their grandmother’s expansive house and yard, and eating plenty of home-cooked meals. As far as trips go, it was simple; there was no jam-packed itinerary, and no sight-seeing—the purpose of the trip was to spend time with family.

Several days before flying back home, Monica developed fever, vomiting, and diarrhea. The clinic near her grandmother’s neighborhood diagnosed her with gastroenteritis and gave her an antibiotic and nausea medicine. Unfortunately, her symptoms decided to progress during the flight home. After landing back in Maryland, her mother took her to their local emergency room, and she was admitted to the hospital for intravenous fluids and antibiotics while they awaited the results of her blood cultures. When her blood cultures grew a gram-negative bacillus, she was transferred to the nearest tertiary care children’s hospital, located in Washington, DC, for treatment of a life-threatening bacteremia.

Monica was soon seen by the pediatric infectious diseases team, who took a thorough travel history. They learned that Monica’s grandmother’s house was 7,000 miles away in Karachi, Pakistan. What was highest on their differential? Typhoid fever.

Caused by several serotypes of *Salmonella enterica* subspecies *enterica*, typhoid fever is a bacterial illness spread through contaminated food and water. Pakistan is no stranger to typhoid fever, with severe outbreaks of the disease occurring as recently as 2023. Poor sanitation and water quality have exacerbated the rapid increase in typhoid cases in urban areas like Karachi. Another threat is the rise of extensively drug resistant (XDR) typhoid in Pakistan, which has rendered many antibiotics obsolete. The pediatric infectious disease team reasoned that there was a high chance that she had contracted typhoid fever. As such, she was started on intravenous meropenem—one of the few antibiotics that could effectively treat an XDR strain.

To their surprise, the blood cultures revealed a serotype of *Salmonella enterica* subspecies *enterica* that causes Non-Typhoidal *Salmonella* (NTS) disease, a more mundane pathogen that can be contracted right here in Maryland. Given this plot-twist, the microbiology department repeated the testing at the behest of the pediatric infectious disease team, confirming that it was indeed an NTS infection. While it was the same species and subspecies of *Salmonella*, a different serotype meant it was not the same story. The meropenem was discontinued, and Monica was eventually transitioned to an oral antibiotic to complete her treatment. NTS is a ubiquitous pathogen. Although Monica had gotten sick during her travel abroad, she could have contracted NTS staying at home as well.

A few days later, Michael developed similar symptoms to Monica, with fever, vomiting, and diarrhea. When his blood cultures revealed a gram-negative bacteremia, he was transferred from his local community hospital to the same tertiary care children’s hospital in Washington, DC, where he was greeted by his sister’s pediatric infectious disease team. Now tasked with diagnosing Monica’s younger brother, they wondered: same story, same *Salmonella*? Despite traveling to the same location and eating the same food, this was not the same *Salmonella*. Michael’s blood cultures grew *Salmonella enterica* subspecies *enterica* serotype Typhi, one of the etiological agents of typhoid fever.

As with Monica’s results, the pediatric infectious disease team requested the microbiology department to repeat the testing of Michael’s isolate – the original result was once again confirmed. One sibling had an NTS infection. The other had typhoid fever. Fortunately, Michael did not have an XDR typhoid strain, and the susceptibility pattern of his isolate allowed for de-escalation and eventual transition to an oral agent to complete his antibiotic course.

It’s not every day that two siblings return after international travel with two remarkably similar and yet profoundly different bacterial illnesses. The global connectivity of the 21st century has changed the rules – disease can cross all borders and boundaries. Is there anything that really makes typhoid fever specifically “tropical?” NTS is present worldwide – could typhoid fever become endemic outside of the tropics? Modern air travel has erased many of the borders that used to confine tropical diseases to, well, the tropics. Climate change and urbanization have created a world in which disease can easily cross continents. The experiences of Monica and Michael illustrate how tropical medicine can be right “here.” When considering tropical medicine in the 21st century, we must be ready to appreciate that the distinctions of “here” versus “there” are less well-defined than ever before, and that tropical medicine may need to be practiced in your local neighborhood.

